# The complete chloroplast genome of *Macrothelypteris torresiana*, a reputed medicinal fern (Thelypteridaceae)

**DOI:** 10.1080/23802359.2018.1501317

**Published:** 2018-08-17

**Authors:** Songyan Zhou, Shuaixi Zhou, Ruixiang Xu, Shanshan Liu, Ziqing He, Zhen Wang, Ting Wang, Yingjuan Su

**Affiliations:** a School of Life Sciences, Sun Yat-sen University, Guangzhou, China;; b College of Life Sciences, Nanjing Agricultural University, Nanjing, China;; c College of Life Sciences, South China Agricultural University, Guangzhou, China;; d Research Institute of Sun Yat-sen University in Shenzhen, Shenzhen, China

**Keywords:** A reputed medicinal fern, chloroplast genome, *Macrothelypteris torresiana*, phylogenetic analysis

## Abstract

*Macrothelypteris torresiana* is a reputed medicinal fern. Its complete chloroplast genome was determined by Illumina paired-end sequencing. The genome is 151,150 bp in length with 43.1% overall Guanine+Cytosine (GC) content, which is divided into four distinct parts such as a small single copy (SSC, 21,772 bp), a large single copy (LSC, 82,422 bp), and two inverted repeats (IRs, 23,478 bp each). It contains 132 genes, including 86 protein-coding genes, eight ribosomal RNA genes, 35 tRNA genes, and three pseudogenes. Maximum likelihood (ML) tree revealed that *M. torresiana* was closely grouped with *Christella appendiculata* with 100% bootstrap value.

*Macrothelypteris torresiana* (Gaud.) Ching is a robust fern belonging to Thelypteridaceae. Its distinguished features include the glaucous-white, almost glabrous young stripes prior to the fronds unfurling (de Lange and Crowcroft 1997). The species prefers wet places in mountain valleys at an altitude of 1000 m with distribution in tropical and subtropical regions including China, America, Australia, and Pacific islands (Lin et al. 2013). As a reputed folk medicinal fern, its leaves and roots are widely used for fever, pain, and granulation in Southeast Asia and for kidney problems in China (Huang et al. 2010; Chen et al. 2012). The species is suffering different classified process including assigning to *Macrothelypteris*, *Phegopteris*, and allied genera (Smith and Cranfill 2002; He and Zhang 2012). Hence, the acquirement of whole chloroplast (cp) genome of *M. torresiana* will be helpful to deeply investigate phylogenetic relationships of Thelypteridaceous genera.

We obtained mature and healthy leaves of *M. torresiana* from South China Botanical Garden, Chinese Academy of Sciences (23°11′3.56″N, 113°21′43.28″E), which was used to DNA extraction through Tiangen Plant Genomic DNA Kit (Tiangen Biotech Co., Beijing, China). The specimen is stored in the Herbarium of Sun Yat-sen University (SYS; voucher: *SS Liu 201618*). After DNA was broken into 300 bp fragment using Covaris M220 (Covaris Inc., Woburn, MA), an Illumina paired-end (PE) genomic library was constructed and genome sequencing was performed on Hiseq 2500 platform (Illumina Inc., San Diego, CA). In total, 2.04 G raw reads were quality-trimmed by Trimmomatic v0.32 (Bolger et al. 2014) and 1.74 G clean data were used to assemble the chloroplast genome by Velvet v1.2.07 (Zerbino and Birney 2008). We used Dual Organellar GenoMe Annotator (DOGMA; Wyman et al. 2004) to annotate genes and tRNAscan-SE programs (Lowe and Eddy 1997) to confirm tRNAs. In order to survey phylogenetic classification, we selected the complete chloroplast genome sequence of 11 ferns including *M. torresiana* with *Angiopteris evecta* as an outgroup and created a multiple sequence alignment by the MAFFT v7.311 (Katoh and Standley 2013). A maximum likelihood (ML) tree was inferred using RAxML v.8.0 (Stamatakis 2014) with 1000 bootstrap replicates.

The complete chloroplast genome of *M. torresiana* is a double-stranded circular DNA of 151,150 bp in length with 43.1% overall GC content (GenBank Accession Number: MH500230), which was divided into four distinct parts such as a small single copy (SSC) of 21,772 bp, a large single copy (LSC) of 82,422 bp, and two inverted repeats (IRa and IRb) of 23,478 bp. The GC content of the LSC, SSC, and IR are 42.6, 40.2, and 45.3%, respectively. The chloroplast genome contains 132 genes, including 86 protein-coding genes, eight ribosomal RNA genes, 35 tRNA genes, and three pseudogenes, and 14 genes duplicated in the IR region. Eighteen genes have one or two introns involving *ndhB*, *rps16*, *atpF*, *rpoC1*, *petB*, *petD*, *ndhA*, *rpl16*, *rpl2*, *trnG-UCC*, *trnV-UAC*, *trnA-UGC*, *trnI-GAU*, *trnL-UAA*, *trnT-UGU*, *ycf3*, *clpP*, and *rps12*. The ML tree showed that *M. torresiana* was closely grouped with *Christella appendiculata* with 100% bootstrap value (Figure 1). The complete chloroplast genome of *M. torresiana* will provide powerful data for facilitating phylogenomics of ferns.

**Figure 1.**
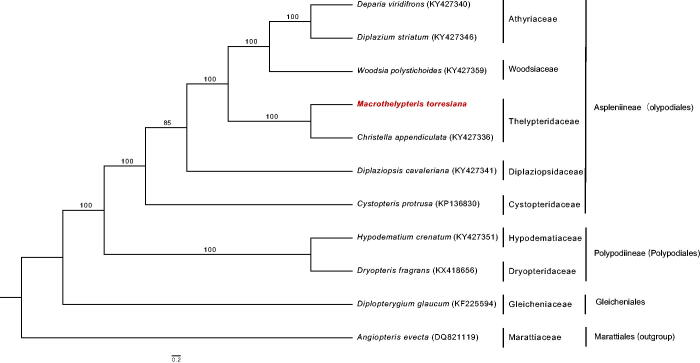
Maximum likelihood phylogenetic tree of *Macrothelypteris torresiana* with 11 ferns including *Angiopteris evecta* as an outgroup based on the complete chloroplast genome. The numbers near the branches are bootstraps values for 1000 replicates.
